# Theoretical Framing and Dissemination of Ageism First Aid: A Significant Tool for the AFU Movement

**DOI:** 10.1093/geroni/igaa057.1728

**Published:** 2020-12-16

**Authors:** Laurinda Reynolds, Tina Newsham

**Affiliations:** 1 American River College, Sacramento, California, United States; 2 University of North Carolina Wilmington, Wilmington, North Carolina, United States

## Abstract

By graduation most high school students have negative feelings about aging, the idea of working with older people is unappealing, and few choose to study aging. When non-traditional students of any age enroll after gaining experience in the workforce, they experience ageism and may not persist to degree completion. The value of Ageism First Aid (AFA) as an internal and external AFU tool is supported by Bronfenbrenner’s Ecological Theory and Rogers’ Diffusion of Innovation theory. AFA utilization during high school can increase traditionally-aged student enrollments. Its use for professional development on campuses can improve the campus climate for all constituents and ensure graduates will know how to avoid contributing to aging stigma. AFA use by providers of aging services and support can reduce the aging stigma older people experience. Attendees will receive handouts with a variety of dissemination models and information on how to gain institutional access to AFA. Part of a symposium sponsored by Age-Friendly University (AFU) Interest Group.

